# International Congress on Automation in the Clinical Laboratory: Editorial

**DOI:** 10.1155/S146392468200025X

**Published:** 1982


Journal of Automatic Chemistry, Volume 4, Number 2 (April–June 1982), pages 90–106

### International Congress on Automation in the Clinical Laboratory: Editorial

The last number of *Journal of Automatic Chemistry* marked the first edition produced by our new publisher, likewise this second issue also marks a new departure. Part of this issue is devoted to the publication of abstracts for the International Congress on Automation in the Clinical Laboratory. This meeting is being held in Barcelona from 19 to 22 April 1982, and the abstracts are devoted to various aspects of automation as applied to the clinical laboratory. However, the problems of applying computers to instrumentation, or to the general work-flow in any laboratory, present similar problems to the user and to the systems designer and there is considerable merit in sharing experiences. Further details on the papers abstracted can be obtained directly from their authors, although I hope that several of them will submit the full text of their papers for publication in the *Journal*. Every participant at the congress will be presented with a copy of the *Journal* with their registration documents. If it is the first time that they have seen the *Journal*, we welcome them as readers and hope that within these pages they will find much of value to their work. I also hope that they have a valuable and enjoyable congress and gain much of value from their participation.

On a personal note, I have just returned from a brief visit to the 33rd Annual Pittsburgh Conference and the large exhibition will be presented in the next issue. There, during a discussion with two members of my main editorial board—Professor Howard Malmstadt and Dr Rolf Arndt—about inviting Professor G. Horlick to the editorial team, a very interesting suggestion was raised. It was proposed that the formation of a society of laboratory automation would be an attractive concept and that it should be directly linked to the *Journal*. This should encourage a broader subscription base for the *Journal* and promote conferences on laboratory automation where clinical chemists and industrial chemists, along with those in academic life, can discuss the many problems involved in introducing computerization and automation into various work environments. The proposal certainly has considerable appeal and I would welcome reactions from our readers. A world-wide conference on laboratory automation, along the lines of the congress in Barcelona, would be a valuable forum for discussing the problems and advantages of automation.

An interesting development at Pittsburgh was the introduction of an automated system based on robotics. The Zymark Corporation was formed in March 81 to develop, manufacture and market instrumentation for automated laboratory-scale processes used in chemistry and biochemistry. The Zymate Laboratory Automation system combines robotics and laboratory stations to automate the procedures used in sample preparation. For example liquid handling, sample conditioning, separation and chemical modification. In addition to the instrumentation, the Zymark Corporation has developed an educational program to provide a foundation in modern techniques of sample preparation—this program is available on a subscription basis. Whilst the approach is not totally new—the Robot Chemist and similar systems were launched more than 10 years ago—the technology now available makes the chances of success of this new venture more likely. Future developments along similar lines are eagerly awaited.

**Peter B. Stockwell**

### Congress Abstracts

### Session 20.1: Clinical Chemistry—General

**20.1.1: A colorimetric alpha-amylase method on automated analysers**

*By J. L. Derocque, Boehringer Mannheim GmbH, Mannheim, FR Germany*

A survey of the various methods for the assay of alpha-amylase activity in serum and urine is presented. Their suitability for application to automated instruments is discussed, and a new kinetic alpha-amylase method using para-nitrophenyl-maltoheptaoside as a chromogenic substrate is described. This method is free from interference from endogenous glucose and can easily be adapted to most of the common automated systems.

An international inter-laboratory survey including more than 1000 laboratories in 14 European countries was carried out. The participants in this survey were requested to report simultaneously the results they obtained with the new method and the results of the method routinely used in their laboratory. Evaluation of the results showed that the inter-laboratory coefficient of variation (CV = 12%), obtained with the new method was good and comparable with those obtained for other enzymes (for example alkaline phosphatase, gamma-GT) in surveys carried out in Germany. The inter-laboratory precision was also far better than that obtained with most of other alpha-amylase methods.

Results are also discussed according to the various instruments used in this survey.

**20.1.2: Determination of serum urea with use of o-phthaldehyde reagent in the Coulter chemistry CA-3 and ABA-100**

*By J. M. Paz, A. Lopez-Urrutia, J. C. Tutor and Del Rio, Laboratorio Central, Hospital General de Galicia, Universidad de Santiago de Compostela, Spain*

The o-phthaldehyde procedure, which was developed by Jung for the measurement of urea, was adapted to the Coulter chemistry CA-3 and ABA-100 and evaluated for accuracy, precision, linearity and sample-to-sample interaction. The results obtained were compared with those for the urease/GLDH and diacetyl methods.

The precision obtained was good (CP $>98{\cdot}5$) and the total analytical error was not medically significant. The linearity throughout was 1·33–44·96 mmol/l. A sample-to-sample interaction was observed only for Coulter chemistry. For 121 random samples diacetyl (Coulter Chemistry) versus o-phthaldehyde (Coulter chemistry) gave a Pearson coefficient ($r$) of 0·998 ($y = 0{\cdot}98 + 0{\cdot}10$). For 63 samples o-phthaldehyde (Coulter chemistry) versus urease/GLDH (ABA-100), $r = 0{\cdot}998$ ($y = 0{\cdot}97x + 0{\cdot}18$). For 75 samples diacetyl (Coulter chemistry) versus o-phthaldehyde (ABA-100), $r = 0{\cdot}999$ ($y = 0{\cdot}99x + 0{\cdot}13$).

The Jung procedure can be simply adapted to the Coulter chemistry and ABA-100. It provides an accurate, precise and economical alternative to the diacetyl and urease/GLDH methods.

